# Anti-Ku Antibodies in a Patient With a Polymyositis-Systemic Sclerosis Overlap Syndrome in Association With Autoimmune Hepatitis

**DOI:** 10.7759/cureus.108420

**Published:** 2026-05-07

**Authors:** Muhammad Zubair, Maha Wazir, Farhan Ullah, Sam J Thomson, Alistair Hepburn

**Affiliations:** 1 Rheumatology, Worthing Hospital, University Hospital Sussex NHS Foundation Trust, Worthing, GBR; 2 Nephrology, The Ohio State University Wexner Medical Center, Columbus, USA; 3 Nephrology, Ochsner Medical Center, Jefferson, USA; 4 Gastroenterology and Hepatology, Worthing Hospital, University Hospital Sussex NHS Foundation Trust, Worthing, GBR

**Keywords:** anti-ku antibodies, autoimmune hepatitis, autoimmune overlap syndrome, polymyositis-systemic sclerosis overlap syndrome, polymyositis with vasculitis

## Abstract

Overlap syndromes are well-described but rare clinical entities characterized by the co-presence of two or more connective tissue diseases. The combination of systemic sclerosis and polymyositis overlapping with autoimmune hepatitis (AIH) is particularly rare, as discussed in this case report. A 76-year-old woman with a recent history of pleuro-pericarditis presented with painful Raynaud’s phenomenon, lethargy, jaundice, myalgias, and weight loss. Blood tests revealed a positive antinuclear antibody and anti-Ku antibody and raised creatine kinase, together with evidence of myositis and interstitial lung disease on imaging, leading to a diagnosis of polymyositis-systemic sclerosis overlap syndrome. Raised liver transaminases, immunological tests, and liver biopsy were consistent with AIH. She was managed with corticosteroids and mycophenolate mofetil. This is only the second instance in which AIH has been reported in the context of a positive anti-Ku antibody. We discussed the complex and rare nature of this condition and the various aspects of its management.

## Introduction

Overlap syndromes refer to autoimmune disorders that satisfy classification criteria for two or more connective tissue diseases [1]. Systemic sclerosis (SSc), a systemic autoimmune condition characterized by vasculopathy and fibrosis of internal organs and skin, is deemed to be the most common overlapping condition with polymyositis (PM) [2]. Common features in polymyositis-systemic sclerosis (PM-SSc) overlap include arthritis, sclerodactyly, interstitial lung disease (ILD), Raynaud’s phenomenon, evidence of muscle inflammation, and the presence of autoantibodies, such as anti-Ku and anti-PM-Scl antibody [3].

In SSc, liver involvement is recognized, with primary biliary cirrhosis being the most frequent association [4,5]. However, concomitant presence of autoimmune hepatitis (AIH) is extremely rare in PM-SSc overlap syndromes [6,7].

Here, we report a case of PM-SSc overlap and in association with AIH, in which the presence of anti-Ku antibodies was detected. To the best of our knowledge, this is only the second instance of an anti-Ku antibody being found in conjunction with the aforementioned overlap connective tissue disorder.

## Case presentation

A 76-year-old woman was admitted from the outpatient Gastroenterology clinic, where she was being investigated for suspected AIH. She presented with a seven-month history of general decline, easy fatigability, myalgia, jaundice, and pruritus. She had anorexia and a corresponding weight loss of 6 kg. However, there were no reports of fever, night sweats, or focal infective symptoms. She was also experiencing arthralgia, muscle weakness, and worsening Raynaud's symptoms for the last 2-3 months.

Two months before the onset of these symptoms, she developed pleuropericarditis. At that time, the pleural fluid cultures were negative, including AFB, so it was deemed post-viral in origin. She was treated with colchicine, with a subsequent radiological and electrophysiological improvement, albeit with slowly resolving pleuritic symptoms.

There was a previous diagnosis of osteoarthritis (OA) of the hands, and primary Raynaud's phenomenon had been diagnosed seven years ago. In addition, she was known to have atrial fibrillation, treated with bisoprolol and apixaban. Previous attempts at cardioversion to sinus rhythm had been unsuccessful. She had never smoked, but consumed alcohol within recommended safe limits.

On initial examination, her blood pressure was 116/71 mmHg, temperature 36.4 °C, and respiratory rate was 19 breaths per minute. Her heart rate was 115 per minute, and this was irregular. Her BMI was low at 18.4 kg/m^2^. She appeared dehydrated clinically with a capillary refill time of four seconds and dry oral mucosa. She was jaundiced, but there was no rash or lymphadenopathy. There were Heberden's nodes in the hands in keeping with nodular OA, but clinically, there was no evidence of synovitis. She had some features of SSc, with mild microstomia and shiny, thickened skin distal to metacarpophalangeal and metatarsophalangeal joints, but there were no digital ulcers or nail fold infarcts. Proximal muscle weakness was detected with MRC power of 4/5 on hip extension, with depressed deep tendon knee reflexes. Abdominal examination was unremarkable, but there were bilateral basal crackles on chest auscultation. Laboratory investigations demonstrated grossly deranged liver function tests with a mixed cholestatic-hepatitic picture, markedly elevated creatine kinase, hypergammaglobulinemia, and hypocomplementemia (Table 1).

**Table 1:** Laboratory findings at presentation FEV1: Forced expiratory volume; FVC: forced vital capacity; TLCO: transfer factor for carbon monoxide

Laboratory Parameter	Result	Reference Range	Status
Complete Blood Count			
Hemoglobin	13.5 g/dL	12.0-15.0 g/dL	Normal
Platelets	376 × 10⁹/L	150-410 × 10⁹/L	Normal
White Cell Count	12.5 × 10⁹/L	4-10 × 10⁹/L	High
Renal Function			
Creatinine	58 μmol/L	50-98 μmol/L	Normal
Urinalysis (Blood)	Negative	Negative	Normal
Urinalysis (Protein)	Negative	Negative	Normal
Inflammatory Markers			
C-Reactive Protein (CRP)	15 mg/L	0-5 mg/L	High
Liver Function Tests			
Bilirubin	72 μmol/L	2-21 μmol/L	High
Alanine Transaminase (ALT)	541 U/L	5-40 U/L	High
Aspartate Aminotransferase (AST)	659 U/L	5-40 U/L	High
Alkaline Phosphatase (ALP)	213 U/L	20-150 U/L	High
Immunoglobulins			
Immunoglobulin G (IgG)	30.5 g/L	5.5-16.3 g/L	High
Muscle Enzymes			
Creatine Kinase (CK)	1474 U/L	25-200 U/L	High
Complement Levels			
C3 Complement	0.55 g/L	0.8-1.9 g/L	Low
C4 Complement	0.1 g/L	0.2-0.6 g/L	Low
Autoimmune Serology			
Anti-Smooth Muscle Antibody (ASMA)	Positive	Negative	Positive
ANA (HEp-2)	Positive (Fine Speckled)	Negative	Positive
Anti-Ku Antibodies	Positive	Negative	Positive
Viral Serology			
Hepatitis B	Negative	Negative	Normal
Hepatitis C	Negative	Negative	Normal
Pulmonary Function Tests			
FEV1	Normal	Normal	Normal
FVC	Normal	Normal	Normal
TLCO (Gas Transfer)	3.48 (55% predicted)	>80% predicted	Low

**Figure 1:**
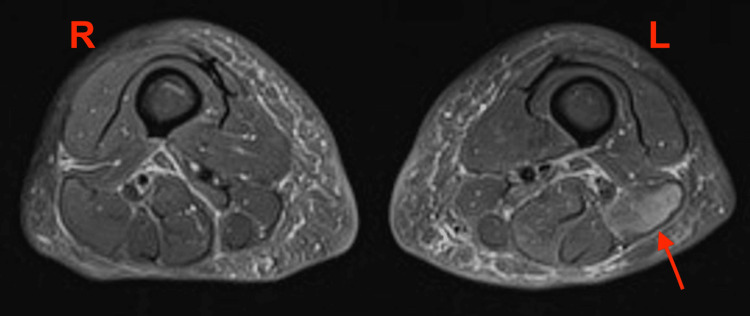
Axial STIR MRI image of both thighs demonstrating a high signal in the biceps femoris on the left suggesting localized myositis STIR: Short tau inversion recovery

**Figure 2:**
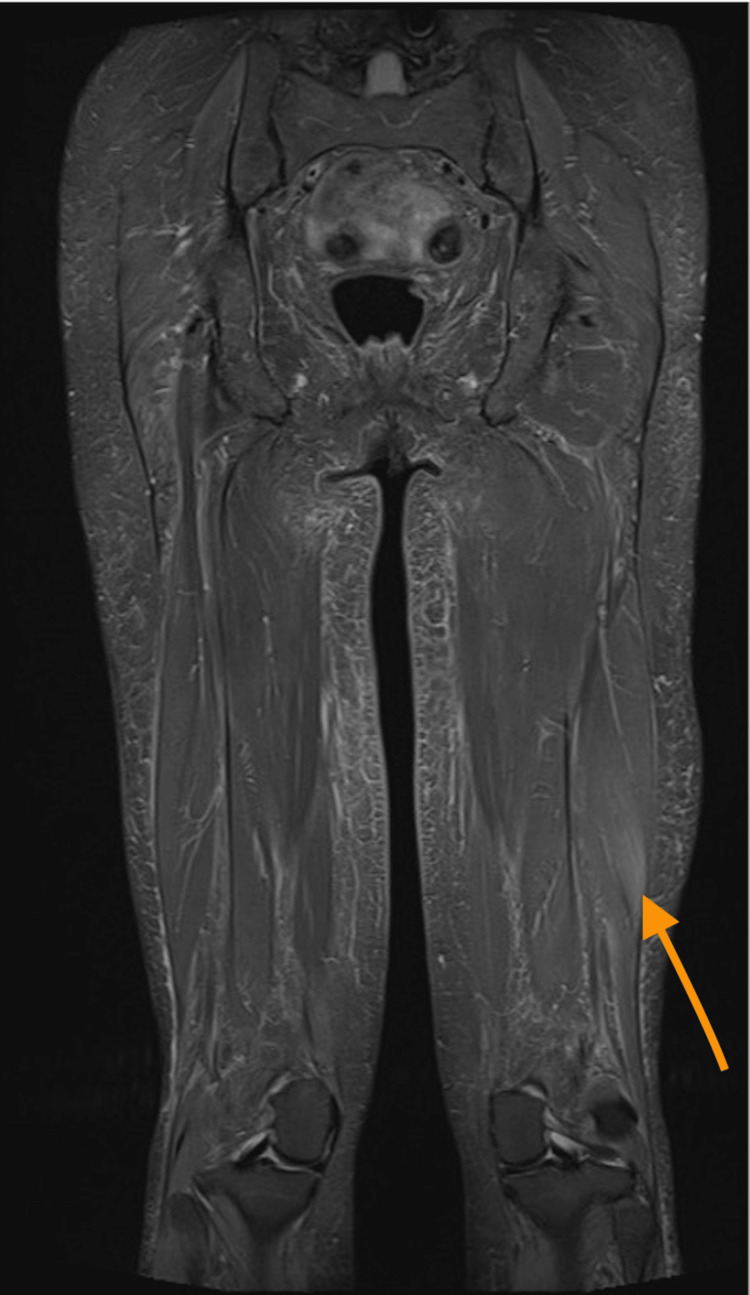
Coronal-section MRI (STIR sequence) of both thighs demonstrating localized myositis in the biceps femoris on the left side STIR: Short tau inversion recovery

The immunology screen revealed a positive anti-smooth muscle antibody (ASMA) and low levels of C3 and C4 at 0.55 g/L (0.8-1.9 g/L) and 0.1 g/L (0.2-0.6 g/L), respectively. ANA, using the HEp-2 assay, showed a positive fine speckled pattern. An extended scleroderma screen was positive for anti-Ku antibodies. An MRI scan of the thighs showed subtle signs of inflammation in keeping with myositis (Figures 1, 2). 

**Figure 3:**
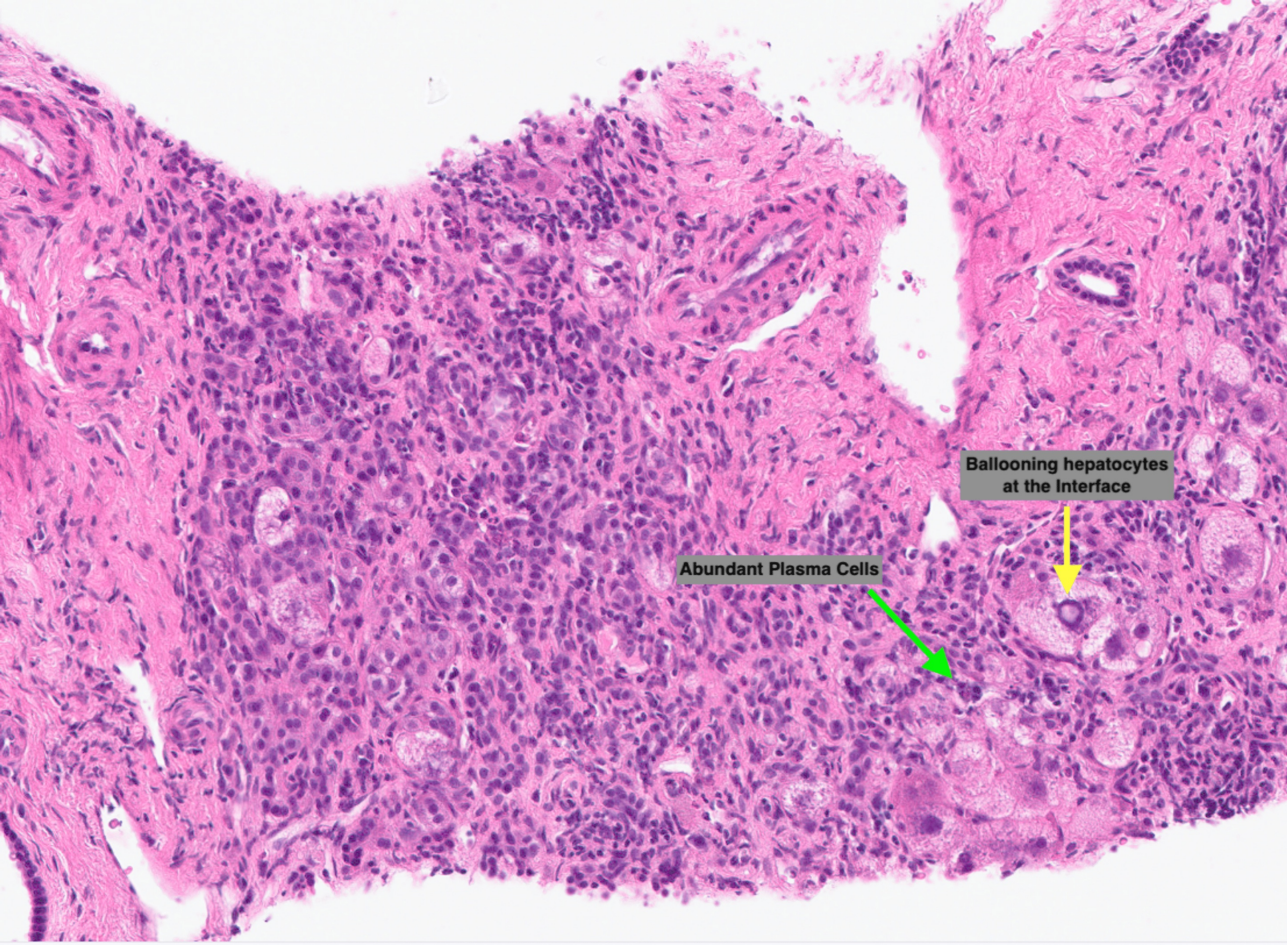
Liver biopsy showing changes consistent with autoimmune hepatitis (AIH) - H&E staining at x43

A transjugular liver biopsy was undertaken, which demonstrated a severe plasma cell-rich hepatitis. The majority of the changes observed were due to collapse rather than fibrosis, which is consistent with AIH (Figure 3). The CT scan of her chest, abdomen, and pelvis was unremarkable, with no signs of malignancy or infection. A barium swallow showed mild reflux disease but no signs of stricture or significant dysmotility. 

**Figure 4:**
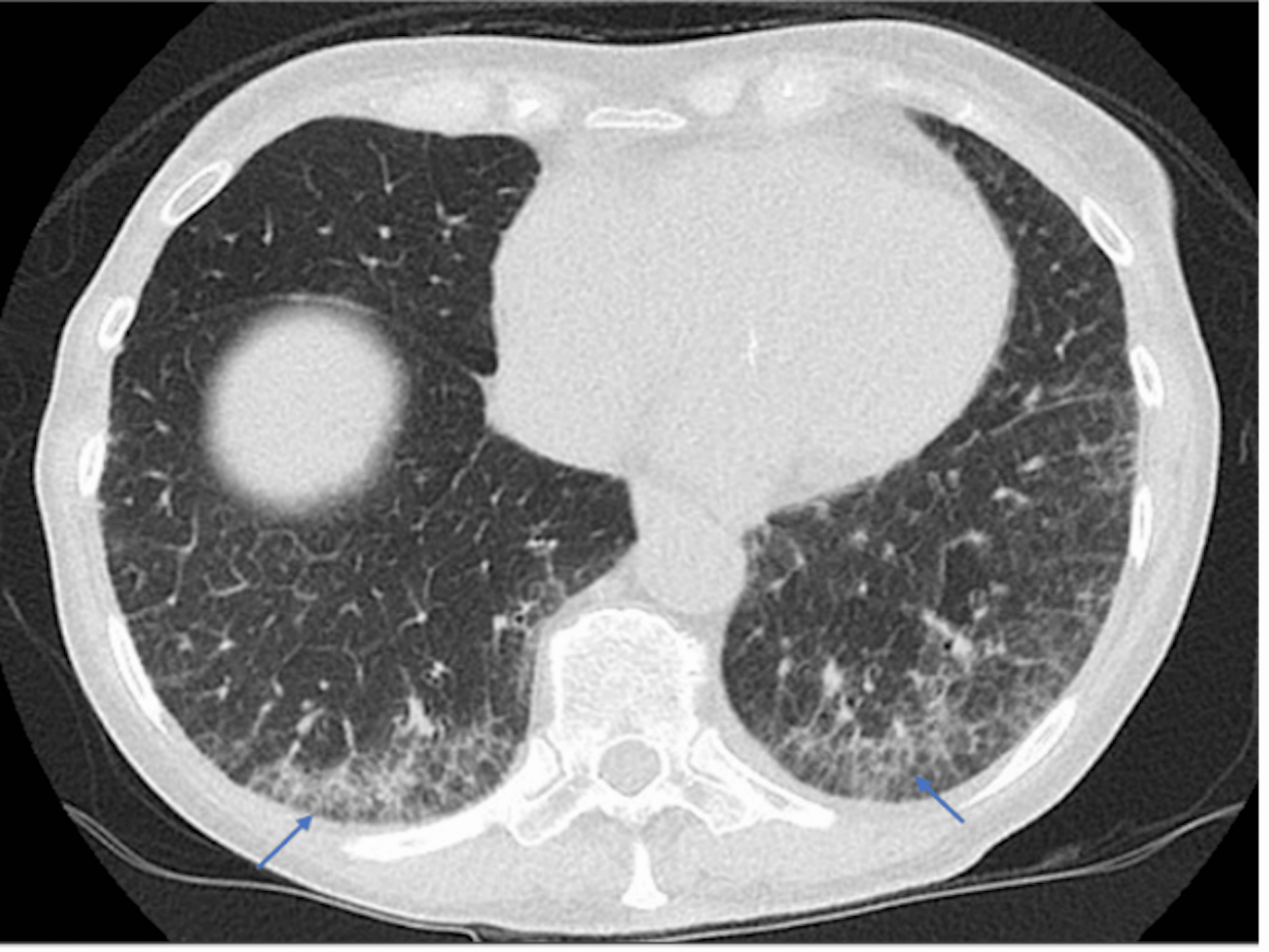
Axial high-resolution computed tomography (HRCT) image of the chest demonstrating interlobular septal thickening and mild subpleural ground-glass opacities in the bilateral lower lobes

**Figure 5:**
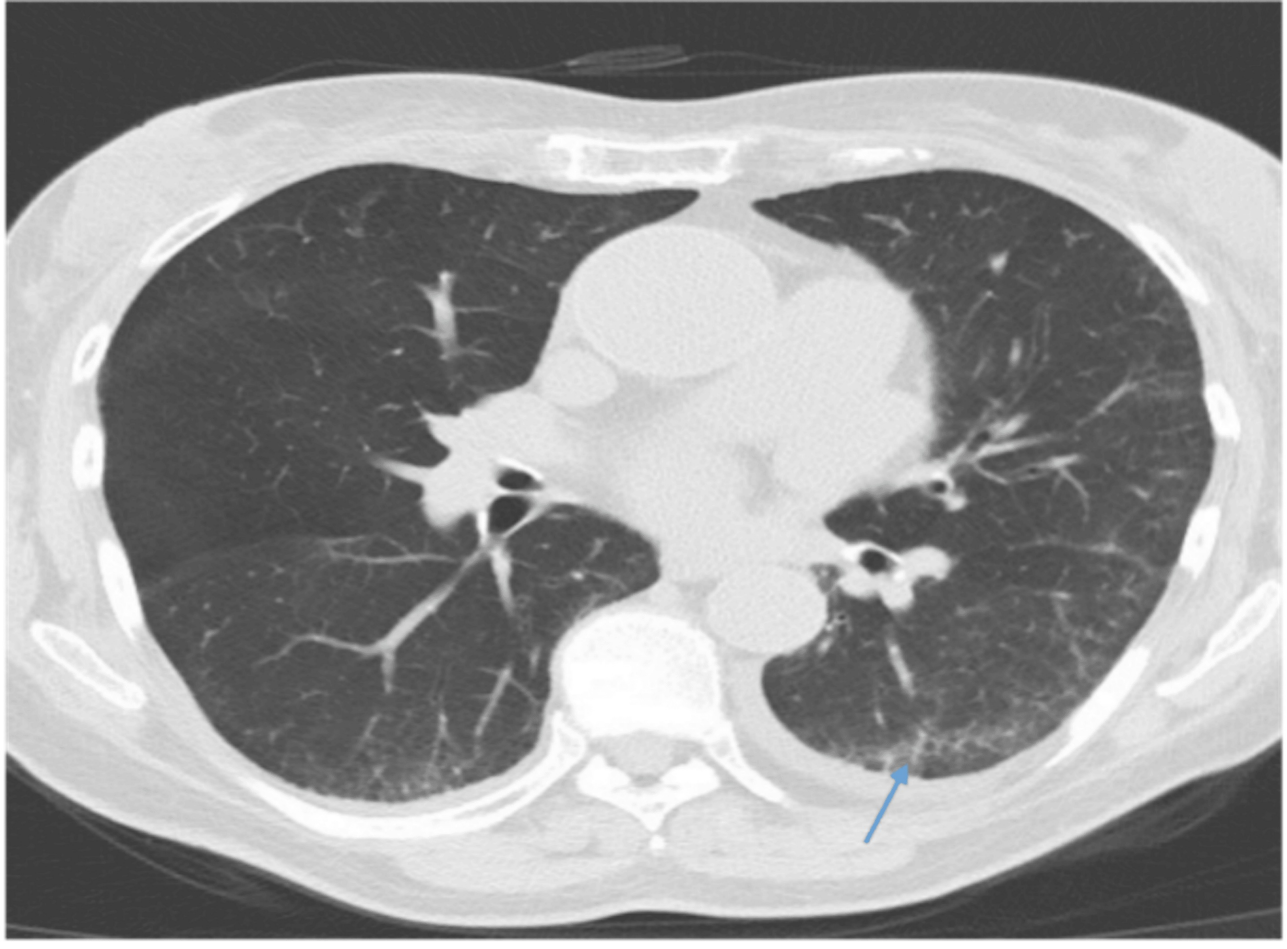
Follow-up axial high-resolution computed tomography (HRCT) chest image after three months of treatment demonstrating near-complete resolution of previously noted septal thickening and ground-glass opacities

The high-resolution CT chest showed early ILD with a nonspecific interstitial pneumonia/organizing pneumonia pattern (Figures 4, 5). Lung function tests showed normal forced expiratory volume (FEV1) and forced vital capacity (FVC), but a low gas transfer TLCO of 3.48 (55% predicted). A transthoracic echocardiogram revealed moderate tricuspid regurgitation and an estimated pulmonary arterial pressure of 26 mmHg.

Using the 2013 ACR/EULAR classification criteria for systemic sclerosis, the patient scored a total of 9 points, meeting the threshold for classification. This was driven by sclerodactyly (4 points), ILD (2 points), and Raynaud’s phenomenon (3 points). Based on the clinical picture, a diagnosis of PM-SSC overlap with associated AIH was made. She was commenced on prednisolone 40 mg/day, weaning by 5 mg/week, with an initial improvement clinically that permitted discharge from the hospital. However, subsequently, she had a recurrence of symptoms with worsening mobility and fatigue upon weaning prednisolone and was thus given three days of IV Methylprednisolone 500mg as an outpatient, to good effect. On her first follow-up visit in the clinic, Mycophenolate mofetil (MMF) 250 mg BD was added as an immunosuppressant, with later dose escalation to 1250 mg BD on subsequent clinic visits. The creatine kinase (CK) and liver function tests (LFTs) completely normalized within the next five months, with a total bilirubin of 10, alanine transaminase of 15, aspartate aminotransferase of 22, and CK of 89. She also experienced marked improvement in her symptoms and overall well-being, although fatigue remains a dominant ongoing symptom.

## Discussion

The initial differential included AIH, based on the mixed cholestatic-hepatitic LFT pattern, elevated IgG, and positive ASMA, but this did not account for the concurrent proximal muscle weakness, markedly elevated CK, MRI-confirmed myositis, Raynaud's phenomenon, and early ILD. Isolated PM was considered but felt less likely given the absence of characteristic cutaneous features alongside Raynaud's phenomenon and sclerodermatous skin changes, while isolated SSc did not adequately explain the degree of muscle involvement and CK elevation. Infectious, malignant, and drug-induced causes of both hepatitis and inflammatory myositis were also considered but deemed unlikely based on the clinical picture and investigations. Ultimately, as no single diagnosis sufficiently explained the multisystem presentation, the constellation of findings supported a systemic connective tissue disease overlap syndrome, with anti-Ku antibody positivity further corroborating a PM-SSc overlap phenotype. AIH is a chronic inflammatory condition of the liver characterized by transaminitis, auto-antibodies, and elevated serum globulin levels [8]. The association of AIH with other hepatic or extrahepatic conditions is well established. Among extrahepatic autoimmune conditions, autoimmune thyroid disease and Sjogren’s disease are the most common [9]. SSc or its overlap variants are rarely associated with AIH, with only a small number of reported cases in the literature [10].

In our case, the patient had a history of late-onset Raynaud’s phenomenon, diagnosed after the age of 60. Though a CTD screen carried out at that time was negative, an extended scleroderma screen or nail-fold capillaroscopy was not undertaken at that point; this perhaps was a missed opportunity to diagnose SSc earlier. This highlights the importance of thoroughly searching for secondary causes in cases of late-onset Raynaud's phenomenon, particularly autoimmune CTD or hematological disease [11]. In this case, a previous episode of pleuropericarditis was also reported. We hypothesize that this was a possible early manifestation of SSc in this case. A literature review suggests that pleuropericarditis, particularly pericardial involvement, can be a common presentation of SSc [12]. An alternative hypothesis, if a viral infection was the cause of pleuro-pericarditis, is that it may have unmasked or triggered an underlying, indolent course of the autoimmune disorder.

Anti-Ku antibody against the Ku antigen, involved in DNA repair, was initially described in patients with PM-SSc overlap syndrome, accounting for up to 55% cases [13]. More recently, anti-Ku antibody has been described in a range of other connective tissue diseases such as SLE, Sjogren’s, ILD, etc. Presence of anti-Ku in the context of myositis has been associated with a favorable outcome [14]. The presence of these antibodies is also associated with GORD and Raynaud’s phenomenon in particular. However, renal, skin, and neurological diseases appear rare. On reviewing the literature, we found a single case report of PM-SSc accompanied by AIH and sarcoidosis with anti-Ku positivity [15]. However, in that case, the autoimmune profile was negative on initial presentation but developed anti-Ku on subsequent testing, suggesting that the case reported here may be unique.

In the context of SSc, corticosteroids should be used with caution, given the concern regarding the development of SSc renal crisis. This can make overlapping syndromes challenging to manage, where corticosteroids are used as first line, for example, in PM and in ILD. High doses of prednisolone are often required during induction treatment of inflammatory myositis. However, it has been theorized that very early in the course of SSc, use of corticosteroids may in fact have a favorable outcome [16].

In terms of immunomodulatory treatment, MMF was preferred as it has been considered to be effective in PM, SSc, and AIH, and has a good safety profile. In practice, MMF is often tolerated better by patients compared to azathioprine. Other drugs that could be utilized in a patient with the form of overlap syndrome reported here include methotrexate, tacrolimus, and IVIg. Thus far in the case reported here, the patient has achieved sustained remission biochemically, and prednisolone has been successfully reduced to 10 mg/day.

## Conclusions

This case represents a rare presentation of anti-Ku antibody-positive PM-SSc overlap syndrome with concurrent AIH. Late-onset Raynaud's phenomenon warrants comprehensive autoimmune screening, including extended antibody panels and nailfold capillaroscopy, as isolated manifestations like pleuropericarditis may herald early systemic sclerosis. Managing such overlap syndromes requires careful balancing of corticosteroid therapy for myositis and AIH against the risk of scleroderma renal crisis.
